# Prediction of Deep Brain Stimulation Outcome in Parkinson’s Disease With Connectome Based on Hemispheric Asymmetry

**DOI:** 10.3389/fnins.2021.620750

**Published:** 2021-10-26

**Authors:** Jingqi Wang, Ruihong Shang, Le He, Rongsong Zhou, Zhensen Chen, Yu Ma, Xuesong Li

**Affiliations:** ^1^School of Computer Science and Technology, Beijing Institute of Technology, Beijing, China; ^2^Department of Biomedical Engineering, Center for Biomedical Imaging Research, School of Medicine, Tsinghua University, Beijing, China; ^3^Department of Neurosurgery, Tsinghua University Yuquan Hospital, Beijing, China; ^4^Department of Radiology, University of Washington, Seattle, WA, United States

**Keywords:** hemispheric asymmetry, functional connectivity, important feature, resting state functional magnetic resonance imaging (rfMRI), Parkinson’s disease, improvement of motor function, subthalamic nucleus deep brain stimulation (STN-DBS)

## Abstract

Parkinson’s disease (PD) is a neurodegenerative disease that is associated with motor and non-motor symptoms and caused by lack of dopamine in the substantia nigra of the brain. Subthalamic nucleus deep brain stimulation (STN-DBS) is a widely accepted therapy of PD that mainly inserts electrodes into both sides of the brain. The effect of STN-DBS was mainly for motor function, so this study focused on the recovery of motor function for PD after DBS. Hemispherical asymmetry in the brain network is considered to be a potential indicator for diagnosing PD patients. This study investigated the value of hemispheric brain connection asymmetry in predicting the DBS surgery outcome in PD patients. Four types of brain connections, including left intra-hemispheric (LH) connection, right intra-hemispheric (RH) connection, inter-hemispheric homotopic (Ho) connection, and inter-hemispheric heterotopic (He) connection, were constructed based on the resting state functional magnetic resonance imaging (rs-fMRI) performed before the DBS surgery. We used random forest for selecting features and the Ridge model for predicting surgical outcome (i.e., improvement rate of motor function). The functional connectivity analysis showed that the brain has a right laterality: the RH networks has the best correlation (*r* = 0.37, *p* = 5.68E-03) between the predicted value and the true value among the above four connections. Moreover, the region-of-interest (ROI) analysis indicated that the medioventral occipital cortex (MVOcC)–superior temporal gyrus (STG) and thalamus (Tha)–precentral gyrus (PrG) contributed most to the outcome prediction model for DBS without medication. This result provides more support for PD patients to evaluate DBS before surgery.

## Introduction

Parkinson’s disease (PD) is a common degenerative disease of the nervous system, including motor symptoms such as retardation, tremor, and muscle rigidity and non-motor symptoms. Subthalamic nucleus deep brain stimulation (STN-DBS) is a widely accepted therapy for PD, especially when the dopaminergic replacement therapy is unsatisfactory (Kleiner-Fisman et al., 2006). This technique was also used in the pallidum and the STN (Benabid, 2003). DBS can significantly reduce the freeing of gait (Xie et al., 2012), tremor, dyskinesia, or postural instability (Weaver et al., 2005) and thereby improve the quality of life. DBS can also reduce the non-motor symptoms (Hwynn et al., 2011) in PD patients. However, DBS could not ensure a significant therapeutic effect on each patient. In spite of a careful patient selection before the operation, some patients may still show limited or no improvement of their motor functions after surgery. On the other hand, DBS surgery is expensive at the moment. Therefore, how to evaluate the possible therapeutic improvement of each patient especially before the surgery is a question that deserves serious consideration.

A growing number of studies suggest that the variability in treatment response may be linked to cortical blood flow changes (Campbell et al., 2008; Quraan et al., 2014). Research showed that cortical blood flow change abnormalities in patients with schizophrenia may be related to the treatment response to stress symptoms (Masuda et al., 2000). Early scalp acupuncture treatment can speed up the cortical blood flow of patients with acute ischemic stroke, thereby promoting the recovery of motor function (Li et al., 2005). Resting-state functional magnetic resonance imaging (rs-fMRI) is related to cortical blood flow, and the method of analyzing rs-fMRI is usually to construct a brain network. Therefore, the brain network is shown to have great potential in predicting the treatment outcome (van Waarde et al., 2015; Kawahara et al., 2017). Human brain networks can be characterized by estimating interregional synchronization of neural function with rs-fMRI. This study focuses on rs-fMRI instead of task-related fMRI data. There are many measurements (small-worldness, clustering coefficient, local efficiency, modularity, and rich-hub) (Kaiser, 2008) to assess information exchange and processing of the human brain networks, which can be measured after the brain network is converted into a weighted graph. Analyses of functional networks could provide complementary insights into brain organization under pathological conditions. For example, compared to healthy controls, PD patients have lower clustering coefficient and local efficiency (Luo et al., 2015b) and have impaired corticostriatal network pathways and related neural circuits (Hacker et al., 2012).

The dopaminergic denervation of the striatum in PD occurs asymmetrically at the beginning and becomes bilateral gradually along with the disease progression (Hornykiewicz, 1966). As such, the accompanying motor dysfunction symptoms (bradykinesia, tremor, and rigidity) usually begin on the side contralateral to the most affected nigrostriatal pathway and later spread to the opposite side. This study of the striatum in PD indicated that the hemispheric asymmetry of functional connectivity is an important factor that may affect brain network organization, which has been demonstrated in healthy subjects (Tian et al., 2011), patients with Alzheimer’s disease and mild cognitive impairment (Yang et al., 2017), and patients with neuropsychiatric disorders (Sun et al., 2015). It has been well documented that the brain network of PD patients tend to show asymmetry (Luo et al., 2015a).

Machine learning as a powerful data-driven method has been widely used in outcome prediction, including outcome of the DBS surgery (Bermudez et al., 2019; Habets et al., 2020). In this paper, we aimed at creating a machine learning model based on functional connectivity profiles to predict the possible improvements of motor function before the DBS surgery. This model could help us pick out the unsuitable patients. More importantly, the machine learning methods may reveal the potential components that limit the effect of the surgery and help physician improve the technique. This study involves the assessment of preoperative rs-fMRI for predicting the DBS-based improvements of motor function as measured by the Unified Parkinson’s Disease Rating Scale (UPDRS-III) score. Four brain networks were constructed for topological analysis, including intrahemisphere connectivity [left-hemispheric (LH) network and right-hemispheric (RH) network] and inter-hemisphere connectivity [homotopic (Ho: the edges link the geometrically corresponding regions in the two hemispheres) network and heterotopic (He: the edges link the geometrically non-corresponding regions in the two hemispheres) network]. The random forest algorithm (Liaw and Wiener, 2002) was used for feature selection, and the Ridge model was used to predict the improvement of motor function after the DBS surgery in each brain (LH, RH, Ho, and He) network. Pearson analysis was performed on the predicted improvement rate and the real improvement rate to obtain Pearson’s *r* and Pearson’s *p*-values, which were used to evaluate the correlation between brain asymmetry and the improvement rate after DBS surgery. In order to study whether this correlation is affected by age and gender, we divided patients into younger and older groups and divided them into male and female groups.

## Materials and Methods

### Participants

This study was approved by the Ethics Committee of Tsinghua University Yuquan Hospital. All participants gave written informed consent. This study included 55 patients whose age ranged from 29 to 77 in Tsinghua University Yuquan Hospital, Beijing, China, who were diagnosed with idiopathic PD according to the UK PD Society Brain Bank criteria. The exclusion criteria included (1) severe suicidal tendency; (2) pregnant or lactating women; (3) a history of physical diseases that can affect the assessment of PD such as intracranial tumors and communicating hydrocephalus; (4) a history of organic brain disorders such as cerebellum injury, neurological disorders such as repeated strokes, severe dementia at an early stage accompanied by memory, language, and behavior disorders, and other psychiatric disorders; and (5) a history of substance abuse, including tobacco, alcohol, or other psychoactive substances. All participants were right dominant based on screening questions.

The DBS surgery outcome was measured in a medication-off condition where levodopa should not be taken 12 h presurgery or postsurgery by the motor section of the UPDRS-III scale (Antonini et al., 2013). This scale was proposed by a team with more than 10 years’ experience. All patients were on levodopa prior to the study. A small number of patients may have mild confusion after taking levodopa for 1 h. These symptoms will disappear within 12 h of stopping the medicine. UPDRS-III is one of the four scales of UPDRS, a clinical scoring system, which is used to judge the motor function of the pre-operation and post-operation of each participant. There are 27 items in UPDRS-III, and each item is divided into a four-level index, from 0 to 4, where 0 is normal and 4 is serious. It is often used to assess the patient’s progress. For the 55 selected PD patients, the UPDRS-III score was measured twice before and 6 months after the DBS surgery. At least two doctors took the measurement and averaged each time. Among them, presurgical scoring and postsurgical scoring were performed routinely, independent of the study and blind to the analysis outcomes. The physicians who program the DBS and the people who measure these scores after surgery were blinded to the analysis. The mean preoperative and postoperative scores were 43.79 ± 11.78 and 15.35 ± 10.67, and all patients had lower scores after surgery, with a lower UPDRS-III score and higher degree of motor function. The improvement of motor function after DBS surgery is assessed by the △UPDRS-III rate = (UPDRS-III_presurgery_ − UPDRS-III_postsurgery_)/UPDRS-III_presurgery_. The mean △UPDRS-III rate of 55 tested PD patients is 65.62% ± 20.48%.

### Surgical Procedure

DBS surgery was performed using a Leksell stereotaxic frame (Elekta AB, Stockholm, Sweden) under local anesthesia. During the surgery, the micro-electrodes and STN-DBS electrodes (PINS L301, Beijing, China) were placed in the left and right hemispheres of the brain. The microelectrodes were used to record, and then STN-DBS were implanted bilaterally for stimulation to evaluate and confirm the site where the best clinical effect can be obtained. After confirming the placement of the lead, a pulse generator (G102R, PINS, Beijing, China) was used to connect the electrodes and was implanted subcutaneously into the right subclavian region. The optimal stimulation patterns with the highest UPDRS score and maximum improvement rate were selected according to the actual situation of the patient.

### Image Acquisition

The rs-fMRI, high-resolution T1-weighted structural MRI data and T2-weighted structural MRI data were acquired on each participant at Tsinghua University using a 3T Philips Achieva rs-fMRI scanner equipped with a 32-channel head coil. Imaging parameters of rs-fMRI were 35 axial slices; repetition time (TR) = 2,000 ms; scan length in time = 8 min; echo time (TE) = 30 ms; flip angle (FA) = 90°; slice thickness = 4 mm; acquisition matrix = 64 × 64; field of view (FOV) = 224 × 224 mm^2^. Imaging parameters of T1-weighted MRI were as follows: TR/TE = 7.46 ms/3.73 ms, FOV = 256 × 256 mm^2^, acquisition matrix = 256 × 256 × 160, slice thickness = 1.0 mm.

The rs-fMRI (8 min) was performed 2–3 days before the operation, and the participants were asked not to take levodopa for more than 12 h before the scanning to keep medication off. Patients were instructed to relax with their eyes closed and to not fall asleep during the scan.

The purpose of the research is to use preoperative images to evaluate postoperative effects, and rs-fMRI can do the prediction and other analyses. Due to the acquisition conditions, only rs-fMRI before DBS surgery can be acquired, so the UPDRS-III score was used to indicate the improvement of the PD patient’s motor function after the DBS surgery. In brief, rs-fMRI before DBS surgery was used to evaluate the improvement rate of motor function after the DBS surgery.

### Image Preprocessing and Brain Network Construction

In the preprocessing of the rs-fMRI data, the first 10 volumes of each participant were discarded to ensure magnetization equilibrium, slice timing was corrected with the first slice, and head motion was corrected by aligning all image volumes with the first volume. In human rs-fMRI data, a 0.01–0.1 Hz band pass filter was commonly used to keep only the interesting frequencies and discard potential noise sources, including the heart rate and respiration rate, which were ∼1.3 and ∼0.2 Hz, respectively. Higher-frequency signals were considered as noise or physiological signals, which are not neuron signal. In order to perform the group analysis, the functional images were co-registered to the same participant’s T1-weighted structural image, which was normalized to the Montreal Neurological Institute template space.

GRETNA software was used to construct the whole-brain functional network (246 × 246) (Wang et al., 2015) for each participant. The nodes of the brain networks come from the brain segmentation based on the Brainnetome Atlas (BNA) (Fan et al., 2016), which parcellated the whole brain into 210 (105 for each hemisphere) cortical and 36 subcortical regions of interest (ROIs). Then the Pearson correlation coefficients of the rs-fMRI signal between each two ROIs were computed to acquire a whole-brain functional network.

**FIGURE 1:**
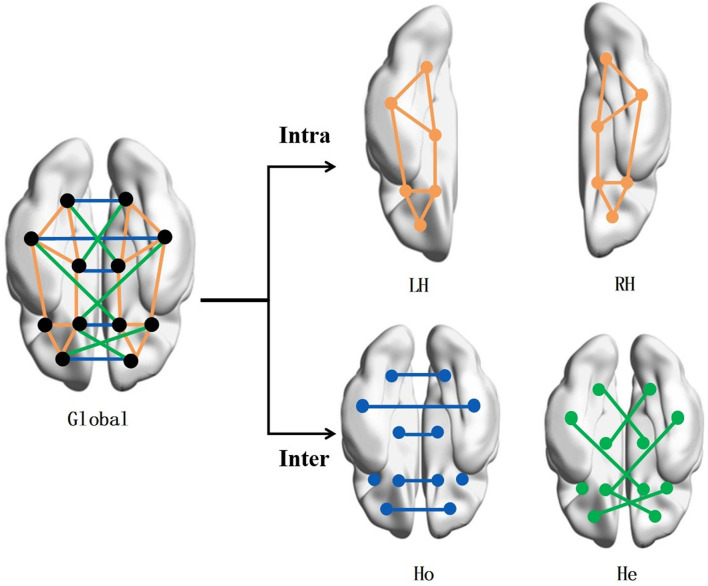
Brain network construction. To study the correlation of brain symmetry on the off-rate of DBS, the brain network is built by intra- and inter-hemispheric connectivity. The connection types include intra-left-hemispheric (LH), intra-right-hemispheric (RH), inter-hemispheric homotopic (Ho), and inter-hemispheric heterotopic (He).

Four brain networks were constructed for topological analysis, i.e., LH network, RH network, Ho network, and He network, as illustrated in Figure 1.

### Connectome-Based Predictive Modeling

For each of the four aforementioned networks (i.e., LH, RH, Ho, and He), the Ridge model was trained to predict the improvement rate of motor function after DBS. For each network, upper triangle elements were used for the input of the Ridge model. Ridge regression has been used as a statistical tool to address the small sample size issue since the 1970s (Hoerl and Kennard, 1970). With the regression coefficient being limited, Ridge regression is free from overfitting and high variances associated with correlated coefficients. Therefore, Ridge regression has many advantages over the traditional multiple-regression models, and it can effectively deal with a large number of predictor variables that are far more than the number of subjects (Li et al., 2006).

Since brain signals are rich and redundant, it is necessary to perform a feature selection strategy to narrow the range of features (i.e., functional connections) for each network. Here, we use the random forest algorithm (Liaw and Wiener, 2002) to select important features for each brain (LH/RH/Ho/He) network. The random forest algorithm is time-efficient in training and can detect the mutual influence between connections as features and therefore is suitable for selecting the most important connections in this study. In this paper, the random forest feature extraction algorithm of the network (LH/RH/Ho/He) is given from the connection information of all cortical region pairs in each network. After the feature vector (upper triangle elements) of the training set was input into the random forest, the importance of each feature (i.e., connection) can be obtained, and only the features with higher importance were retained. Then the retained feature set was applied into the testing set.

The random forest algorithm in this paper was used to extract d times from the d features of the LH, RH, Ho, and He networks with replacement, obtain a sample set, and input it into a decision tree, which is repeated 20 times. In this way, 20 sample sets were each input into 20 decision trees. In the extraction process, the data that were not extracted each time were used as out-of-bag data (OOB). This part of the data can be used for the screening of important features: first, the OOB data error (errOOB1) was calculated, and then noise was added randomly to the feature X of all samples of the OOB data to calculate the OOB error (errOOB2) with noise. The importance of the feature was assessed by $\text{X}=\sum \frac{\left(\mathrm{errOOB2}-\mathrm{errOOB1}\right)}{20}$, and then features with an importance higher than 0.005 were selected. The selected features were less affected by noise, and the prediction was more stable. In the Ridge model, for each topic, we used the percentage UPDRS improvement score and the numerical connection values of the above-mentioned cortical region pairs found from the random forest search (i.e., weights). These were used to train the classifier in a nested leave-one-out cross-validation (LOOCV) approach. And the “leave one” here meant leaving a subject in the training-and-test split. The leave-one-out method was also used for group analysis.

We adopted nested cross-validation including an inner fivefold cross-validation and outer LOOCV to measure prediction accuracy. The structural risk minimization of the Ridge model was equal to the sum of the loss function and regularization (L2 norm). The optimal solution was obtained when the loss function value was as small as possible. The inner fivefold cross-validation was used to determine the optimal parameters (e.g., α and max_iter) for the Ridge model by the grid search method, and LOOCV was used to evaluate the generalizability of the model. α balances the relationship between the two parts of the $J\,\left(\theta \right)=M\,S\,E\,\left(y,\overset{⌢}{y};\theta \right)+\alpha \,\frac{1}{2}\,\sum_{i=1}^{n}\theta_{i}^{2}$ in the loss function (MSE was the mean square error, which was a function of network performance), so that the error is as small as possible, and max_iter is the number of repetitions of the process of calculating the loss function of the full sample and performing a unified gradient update. The R-squared value of the Ridge formulation is slightly lower than that of ordinary regression analysis, but the significance of the regression coefficient is often significantly higher than that of ordinary regression. The R-squared value is between 0 and 1. The closer to 1, the better the fitting effect. In the four networks, the R-squared value of LH is 0.67, the R-squared value of RH is 0.72, the R-squared value of Ho is 0.64, and the R-squared value of He is 0.5. The features come from a vector of (N,d), where N is the number of people and each person is a d-dimensional vector. This d-dimensional vector comes from the network (LH, RH, Ho, and He) of each person. There are d column vectors that are d features. The importance features are selected by random forest. The selected features and actual △UPDRS-III rate of patients were used as input in each training procedure of inner and outer loops. The weighting of each feature (i.e., connection) was determined via the model training and then used to predict the △UPDRS-III rate of patients. Then, two indicators (i.e., Pearson’s *r* and Pearson’s *p*) were utilized to measure the performance of predicting the model in each network.

### Group Analysis

The age of the participants ranged from 29 to 77 (mean age = 58.15 ± 10.13) years. Studies have indicated age-based variations in rs-fMRI networks such that in 20–80-year age groups, some aspects of sensory and cognitive resting state networks show weakening with age (Varangis et al., 2019). To study the effect of age and gender on the correlation between brain asymmetry and the improvement of motor function after DBS surgery, we grouped patients by age and gender. There were 53 participants in the group analysis, excluding one patient with incomplete data and a 29-year-old patient to narrow the age span (previously 55 patients). The 53 patients were divided into two groups as evenly as possible according to age: 26 people aged 34–58 and 27 patients aged 59–77. Also, these 53 patients were divided into two groups according to gender: 29 males and 24 females. We used the leave-one-out method on feature selection and the classifier for age and gender group analyses. Inter-group feature selection was performed instead of intra-group. The *r-* and *p*-values obtained by Pearson analysis were used as the evaluation of correlation.

## Results

### Predicting Improvement in Motor Function After DBS

**FIGURE 2:**
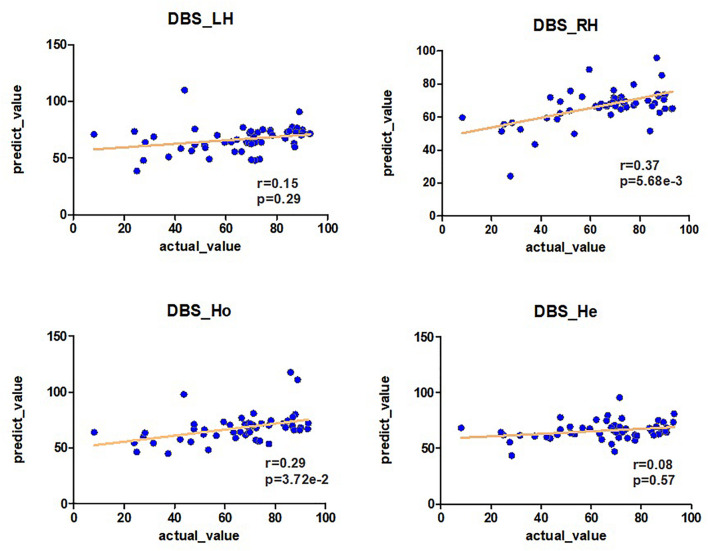
The predictability of brain connection on the DBS off-rate. Pearson analysis is used between the off-rate of DBS and the prediction of brain connections LH, RH, Ho, and He (*r*, *p*). The prediction is obtained by the Ridge model.

The *r-* and *p*-values were observed in the LH network (*r* = 0.15, *p* = 0.29), RH network (*r* = 0.37, *p* = 5.68E-03), Ho network (*r* = 0.29, *p* = 3.72E-02), and He network (*r* = 0.08, *p* = 0.57) as shown in Figure 2 and Table 1. The above results were obtained by using the leave-one-out method in both the feature selection and the classifier.

**TABLE 1:** The top 10 connections in LH **(A)** and RH **(B)** with higher importance in the prediction of the improvement rate in the UPDRS-III score after DBS surgery in the medication-off condition.

(A)				
**ID**	**Node name**	**ID**	**Node name**	**LH normalized connection value**

18	Cingulate gyrus (CG)	18	Cingulate gyrus (CG)	1
17	Insular gyrus (INS)	4	Orbital gyrus (OrG)	0.9
14	Inferior parietal lobule (IPL)	4	Orbital gyrus (OrG)	0.8
18	Cingulate gyrus (CG)	1	Superior frontal gyrus (SFG)	0.7
18	Cingulate gyrus (CG)	14	Inferior parietal lobule (IPL)	0.6
16	Postcentral gyrus (PoG)	15	Precuneus (PCun)	0.5
3	Inferior frontal gyrus (IFG)	11	Parahippocampal gyrus (PhG)	0.4
14	Inferior parietal lobule (IPL)	4	Orbital gyrus (OrG)	0.3
2	Middle frontal gyrus (MFG)	9	Inferior temporal gyrus (ITG)	0.2
23	Basal ganglia (BG)	3	Inferior frontal gyrus (IFG)	0.1

**(B)**				

**ID**	**Node name**	**ID**	**Node name**	**RH normalized connections value**

19	Medioventral occipital cortex (MVOcC)	7	Superior temporal gyrus (STG)	1
24	Thalamus (Tha)	5	Precentral gyrus (PrG)	0.9
11	Parahippocampal gyrus (PhG)	5	Precentral gyrus (PrG)	0.8
23	Basal ganglia (BG)	1	Superior frontal gyrus (SFG)	0.7
7	Superior temporal gyrus (STG)	13	Superior parietal lobule (SPL)	0.6
10	Fusiform gyrus (FuG)	5	Precentral gyrus (PrG)	0.5
24	Thalamus (Tha)	14	Inferior parietal lobule (IPL)	0.4
17	Insular gyrus (INS)	5	Precentral gyrus (PrG)	0.3
23	Basal ganglia (BG)	14	Inferior parietal lobule (IPL)	0.2
24	Thalamus (Tha)	8	Middle temporal gyrus (MTG)	0.1

*1–6: Frontal; 7–12: Temporal; 13–16: Parietal; 17: Insular Lobe; 18: Limbic Lobe; 19, 20: Occipital Lobe; 21–24: Subcortical Nuclei.*

For group analysis (age and gender), we used the leave-one-out method on both feature screening and classifiers. Inter-group feature screening was performed. Among the two groups divided by age mentioned by the participants, the results were as follows: *r* = 0.41, *p* = 0.04 for the younger group and *r* = 0.4, *p* = 0.04 for the older group in the LH network; *r* = 0.58, *p* = 1.35E-03 for the younger group and *r* = 0.43, *p* = 0.02 for the older group in the RH network; *r* = 0.42, *p* = 3.37E-02 for the younger group and *r* = 0.09, *p* = 0.66 for the older group in the Ho network; and *r* = 0.42, *p* = 2.9E-02 for the younger group and *r* = 0.34, *p* = 8.66E-02 for the older group in the He network as shown in Supplementary Table 1A. Patients were also grouped by gender (male and female), the results were as follows: *r* = 0.49, *p* = 8.71E-03 for the male group and *r* = 0.16, *p* = 0.47 for the female group in the LH network; *r* = 0.59, *p* = 7.18E-04 for the male group and *r* = 0.47, *p* = 1.5E-02 for the female group in the RH network; *r* = 0.21, *p* = 0.33 for the male group and *r* = 0.26, *p* = 0.18 for the female group in the Ho network; and *r* = 0.10, *p* = 0.60 for the male group and *r* = 0.69, *p* = 2.1E-04 for the female group in the He network as shown in Supplementary Table 2A. The comparisons of the results obtained by inter-group and intra-group feature selection in age and gender groups were shown in Supplementary Tables 1, 2.

**FIGURE 3:**
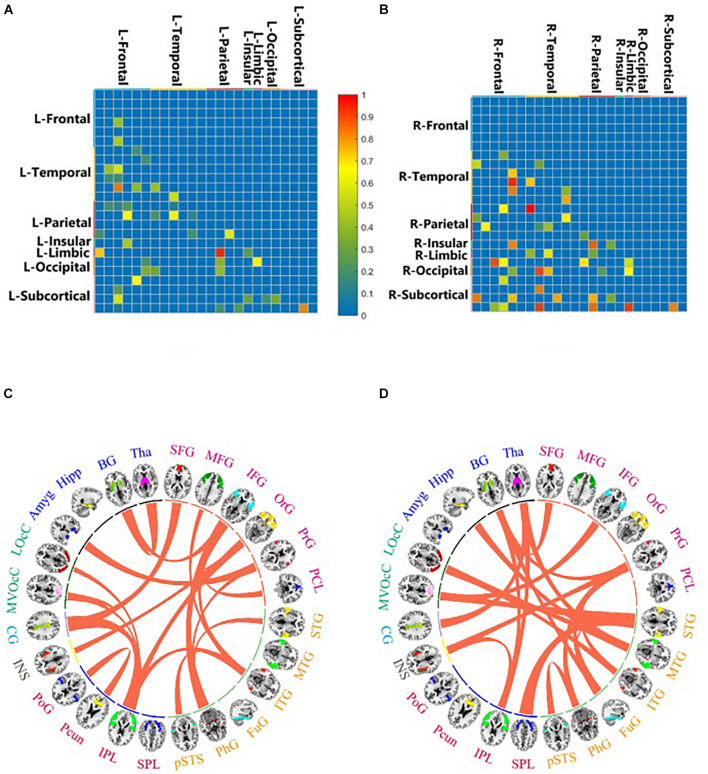
Important brain connections by feature selection of LH **(A,C)** and RH **(B,D)**. The value is the important connection corresponding to the important feature selected by random forest. It ranges from 0 to 1, which is proportional to the importance of the brain connection. Important brain connections by prediction of LH **(C)** and RH **(D)**. This value is the important connection obtained by using the Ridge model for prediction. The important connections between 24 brain areas are connected by curves.

For better interpretation, the four subcortical areas listed were lateralized. We grouped the 246 ROIs into 48 gyri (24 left gyri and 24 right gyri) defined by BNA and calculated the top 10 predictive connections between 24 hemispherical gyri in the LH/RH network. The gyri of each brain hemisphere were further divided into seven lobes, and the predictive connections selected by the Ridge model from the perspective of lobes are shown in Figure 3.

**FIGURE 4:**
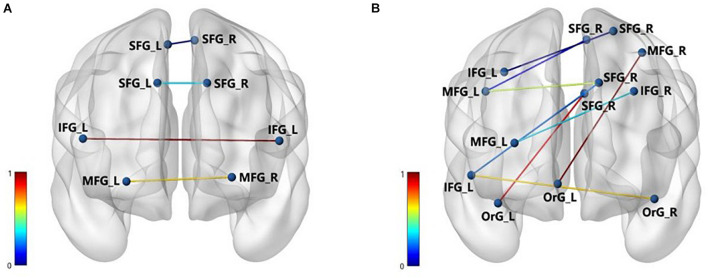
The important brain connections of Ho **(A)** and He **(B)**. The nodes represent the corresponding brain area, and the edges are the important connection between the areas. The normalized values of the edges indicate the importance of brain connection.

In predicting the DBS outcome, the connectome-based model showed the best correlation between the predicted value and the true value in the RH network, and the connection between medioventral occipital cortex (MVOcC) and superior temporal gyrus (STG) provided the largest contribution in the prediction. The top 10 predictive connections (measured by *r*) of the LH network and RH network are shown in Table 2, and the predictive connections of the Ho network and He network are shown in Figure 4.

**TABLE 2:** The correlation of the four networks for the improvement of motor function after DBS surgery.

	LH	RH	Ho	He
*r*, *p*	*r* = 0.15, *p* = 0.29	*r* = 0.37, *p* = 5.68E-03	*r* = 0.29, *p* = 3.72E-02	*r* = 0.08, *p* = 0.57

## Discussion

In this study, we combine an rs-fMRI graph-based network with a machine learning prediction model to predict the DBS outcome based on brain hemispheric asymmetry. Previous studies on the DBS of PD mainly focused on the accuracy of surgery such as the Lead-DBS v2 method with precise electrode positioning (Horn et al., 2019), stimulation such as closed-loop DBS (Rosin et al., 2011), and treatment such as an omics method for prospective targeted therapy for refractory depression (Rivaposse et al., 2018), etc. In addition, research involving the prediction of brain asymmetry in PD patients had different statistical indicators from this study that were based on the value of voxel-mirrored homotopic connectivity to assess the asymmetry of the hemispheric function and its morphology (Gan et al., 2020), and there were also different prediction methods from this study using the human connectome as a connectivity profile (Horn et al., 2017). The brain asymmetry study that similarly uses machine learning was the analysis of depression (Jiang et al., 2019), not an analysis of PD.

On the basis of stable prediction, further brain analysis could be conducted assisted by this preoperative predicting model. We were able to characterize networks associated with the outcome of DBS surgery therapy in 55 PD patients. Our findings might provide a potential neural biomarker that can detect the hemispheric asymmetry in brain networks for predicting the DBS outcome before surgery.

From the perspective of asymmetry, the degree of intra-nodes and inter-hemispheres reveals that network asymmetry is widely distributed in the human brain functional network. At the functional connection level, the DBS operation recovery of PD showed a rightward advantage in the brain. The RH and Ho networks had significantly predictive effects. It was also found that the symptoms of PD such as hallucinating, dreaming, and frequent dozing may be related to right-hemisphere dysfunction (Stavitsky et al., 2008), and late-stage PD patients exhibited greater atrophy in the bilateral occipital and right-hemisphere−predominant cortical areas (Claassen et al., 2016). Reduced structural connectivity in the right hemisphere of PD patients was also found with freezing of gait (Fling et al., 2013). STN-DBS with two electrodes provided the opportunity to modify stimulation parameters for each hemisphere (Lizarraga et al., 2017), which may alleviate the hemispheric asymmetry in PD patients.

In two groups divided by age, the results of inter-group feature selection showed that all four networks (LH, RH, Ho, and He) of the older group had a low correlation between the predicted value and the true value on the improvement of motor function after DBS surgery. The correlation of the RH network in both groups was higher than that of the LH network reflected by *r*-, *p*-values, and the contrast for laterality for the RH network was much clearer for the older group. As for two groups divided by gender, the RH network was most predictive in the male group, and the He network was most predictive in the female group, which significantly correlated with the improvement rate of DBS surgery in participants. Both male and female groups had significant laterality in the RH network. The male group had higher *r*-values in the LH and RH networks compared with the female group, while the female group had higher *r*-values in the Ho and He networks compared with the male group.

The connection between the MVOcC-STG and thalamus (Tha)–precentral gyrus (PrG) had the greatest contribution to the prediction of surgical outcome in the predictive model based on the RH network. It was reported that MVOcC was metabolically and structurally altered in PD (Ellmore et al., 2020). Compared with that in the HC group, STG exhibited significant reduction of nodal efficiency in PD patients with mild cognitive impairment (Wang et al., 2019), and the functional connectivity between left supramarginal–STG (Wiesman et al., 2016) in PD patients was reduced. The change in activity of Tha neurons in the motor circuits was identified as the most marked differences in PD (Halliday, 2009), and Tha was also considered as a suitable stimulation position for PD patients (Caparroslefebvre et al., 1994), which was centrally located in the pathway of the model of the basal ganglia motor circuit and can inhibit movement (Alexander et al., 1991; Kocabicak et al., 2012). The fibers from cerebellar deep nuclei to PrG were implicated in speech deterioration of PD patients (Fenoy et al., 2016). These ROIs are related to the symptoms of PD, indicating the effectiveness of the predictive model.

In the Ho and He networks, the top 10 predictive connections (measured by *r*) were all distributed in the inferior frontal gyrus (IFG), middle frontal gyrus (MFG), and superior frontal gyrus. These results indicated that the frontal lobe of the Ho and He networks played an important role in predicting the DBS outcome. It was widely accepted that the changes in cognitive function in PD were most closely related to the frontal lobe (Obeso et al., 2012). For the brain functional connection, the connections between the dorsolateral prefrontal cortex (DLPFC) and the IFG, superior frontal gyrus (SFG), and MFG in PD patients were significantly reduced (Dong et al., 2020). The frontal lobe “N30” status indicated the severity of PD movement and can effectively respond to dopamine deficiency (Claassen et al., 2016). “N30” resulted from distinct oscillating and phasic generators in the frontal cortex, and the “N30” component of somatosensory evoked potentials has been recognized as a crucial index of brain sensorimotor processing and has been increasingly used clinically (Cebolla et al., 2011). From the perspective of brain biomarkers, the accumulation of Lewy body in the frontal lobe was related to the risk of PD (Crane et al., 2016). In terms of the improvement rate of UPDRS-III for PD patients, the frontal cortex thickness and cortical atrophy in the frontal lobe may be an obvious predictor of poor prognosis of PD patients after STN-DBS (Muthuraman et al., 2017).

In the LH network, the top 10 predictive connections (measured by *r*) were almost distributed in the inferior parietal lobule (IPL), postcentral gyrus (PoG), and precuneus (Pcun), which indicated that the parietal lobe of the LH network may show great correlation for predicting the outcome after DBS. Previous studies have shown that PD-related cognitive patterns (PDCPs) were characterized by reduced metabolism in the frontal and parietal regions (Huang et al., 2007), which also confirmed the above-mentioned correlation between the frontal lobe of the He and Ho networks in PD patients. For the motor sequence learning task for PD, it was found that a longitudinal decline in activation was related to learning of motor function in the parietal lobe region (Carbon and Eidelberg, 2006). In the case of ON and OFF STN-DBS, gait images can induce activity in the auxiliary movement area and the upper right parietal lobule (Weiss et al., 2015).

There are several limitations in our study. Firstly, the sample size is relatively small for machine learning techniques, which may limit the predictive performance of the generated model. Besides, the existing asymmetry in the cortical structure may bring bias to the experiment. Although symmetrical templates were used, the influence of methodological asymmetry cannot be completely eliminated.

## Conclusion

In this study, we predicted the DBS outcome based on brain hemispheric asymmetry in 55 PD patients. By using random forest to select the important connections and the Ridge model with suitable parameters to predict the improvement rate in UPDRS-III, we proved that the RH network can better predict the improvement rate among the four intra- and inter-brain networks (LH, RH, Ho, and He). Besides, the ROI analysis showed that MVOcC-STG and Tha-PrG of the RH network contributed most in predicting the improvement rate in DBS surgery in the medication-off condition, which has a clinical significance for the presurgical analysis of DBS.

## Data Availability Statement

The raw data supporting the conclusions of this article will be made available by the authors, without undue reservation.

## Ethics Statement

The studies involving human participants were reviewed and approved by the Ethics Committee of Tsinghua University Yuquan Hospital. The patients/participants provided their written informed consent to participate in this study. Written informed consent was obtained from the individual(s) for the publication of any potentially identifiable images or data included in this article.

## Author Contributions

RS and JW conduct experiments and wrote the manuscript. LH: search information and conduct experiments. RZ conduct experiments and get the original data set. ZC participate in the writing and revision of the manuscript. YM conduct experiments, obtain original data set, and supervise the completion of the project. XL determine the subject, participate in experimental design, and assist in writing the manuscript. All authors contributed to the article and approved the submitted version.

## Conflict of Interest

The authors declare that the research was conducted in the absence of any commercial or financial relationships that could be construed as a potential conflict of interest.

## Publisher’s Note

All claims expressed in this article are solely those of the authors and do not necessarily represent those of their affiliated organizations, or those of the publisher, the editors and the reviewers. Any product that may be evaluated in this article, or claim that may be made by its manufacturer, is not guaranteed or endorsed by the publisher.
