# Supplementary material for: Prediction of Deep Brain Stimulation Outcome in Parkinson’s Disease With Connectome Based on Hemispheric Asymmetry
Source: Front Neurosci. 2021 Oct 26;15:620750. doi: 10.3389/fnins.2021.620750 (PMC8576048; doi:10.3389/fnins.2021.620750)
Supplement: Supplementary file 1 [file Data_Sheet_1.docx]

**Supplementary**

1. **Age**

There were 53 participants, excluding one patient with incomplete data and a 29-year-old patient that to narrow the age span (previously 55 patients). The 53 patients were divided into two groups as evenly as possible: 26 people aged 34-58, and 27 patients aged 59-77. The results after dividing groups by age were shown in Table 1. The results of inter-group feature screening showed that all four networks (LH,RH,Ho,He) of the older group had a low correlation between the predicted value and the true value on the improvement of motor function after DBS surgery. The correlation of RH network in both groups was higher than LH network reflected by r, p values, and the contrast for laterality for RH network was much clearer for the older group.

**Table 1. The correlation of the four networks in the two age groups for the improvement of motor function after DBS surgery**

| Age | LH | RH | Ho | He |
| --- | --- | --- | --- | --- |
| 34-58 | (r=0.41, p=0.04) | (r=0.58, p=1.35E-03) | (r=0.42, p=3.37E-02) | (r=0.42, p=2.9E-02) |
| 59-77 | (r=0.4, p=0.04) | (r=0.43, p=0.02) | (r=0.09, p=0.66) | (r=0.34, p=8.66E-02) |

1. **Inter-group feature screening**

| Age | LH | RH | Ho | He |
| --- | --- | --- | --- | --- |
| 34-58 | (r=0.25, p=0.21) | (r=0.37, p=0.06) | (r=0.45, p=1.93E-02) | (r=0.35, p=7.67E-02) |
| 59-77 | (r=0.01, p=0.98) | (r=0.11, p=0.60) | (r=0.28, p=0.16) | (r=0.27, p=0.17) |

1. **Intra-group feature screening**
2. **Gender**

Among the 53 participants, there are 29 males and 24 females. The results after dividing the male and female groups were shown in Table 2. Both male and female groups had significant laterality in RH network. The male group had higher r-values in LH and RH networks, while the female group had higher r-values in Ho and He networks.

**Table 2. The correlation of the four networks in the two gender groups for the improvement of motor function after DBS surgery**

| Gender | LH | RH | Ho | He |
| --- | --- | --- | --- | --- |
| male | (r=0.49, p=8.71E-03) | (r=0.59, p=7.18E-04) | (r=0.21, p=0.33) | (r=0.10, p=0.60) |
| female | (r=0.16, p=0.47) | (r=0.47, p=1.5E-02) | (r=0.26, p=0.18) | (r=0.69, p=2.1E-04) |

1. **Inter-group feature screening**

| Gender | LH | RH | Ho | He |
| --- | --- | --- | --- | --- |
| male | (r=0.12, p=0.53) | (r=0.19, p=0.32) | (r=0.01, p=0.97) | (r=0.29, p=0.17) |
| female | (r=0.26, p=0.22) | (r=0.12, p=0.56) | (r=0.01, p=0.95) | (r=0.31, p=0.1) |

1. **Intra-group feature screening**
2. **co-morbidities**

There were age, course and dosage of patients with co-morbidities:

**Table 3.** The age, course and dosage of patients with co-morbidities

| age | co-morbidities | course of disease | dose |
| --- | --- | --- | --- |
| 51 | hypertension | 15 | 283.67 |
| 65 | hypertension、[hyperglycaemia](http://www.baidu.com/link?url=JzJbQY2M6Z4BKsjKoGqNkZe1wJ39HOJq5xUKvt_Qr0-g6ednA6h-4jqSfL6BXiyD4tt5xAzopiV5cOBa3EUEoNC47wH9Q354LfUuXrDN64z9yiRhrBrVOKof_Ab1a9fW)、fatty liver | 7 | 1025 |
| 64 | type 2 diabetes | 13 | 600 |
| 68 | [coronary heart disease](http://www.baidu.com/link?url=52nEjbM7Tuqz6LsuIIc0qJdmJV2LhCZWr2SXUPCNIVwu-BA-fEI4vNaxi1tzCVMPOwwfKRDANcKwvgq6v8G1FiTNLfWOO9r7AFBzjCNE65Ajzah_01FqPzavP55L9rTv)、hypertension、diabetes | 10 | 525 |
| 58 | [constipation](http://www.baidu.com/link?url=tBwyXADAG0XuAbBhpgFEHwEvozJuObjmJLQ2FLvx3tOPnrwq3aOFCxVM2AHV2Yj1j07TUpij7zZdSQ2nlJ8fB-QArHr89ZpvZk_l9yowzvK)、hemorrhoids | 12 | 700 |
| 48 | [Hypothyroidism](http://www.baidu.com/link?url=KaMxdXsGB5_ZGl4hTjl1Tmxj1siZGrU9ZOoiy3hQCOeJTUzKnN4QwFAL3LClVjZV6UuHgrNKNP09kmfVqV1qK1-M8VEM_Maedd_l-gW33NmdGfuC94O1H_kyoe9Fs-g1) | 6 | 450 |
| 69 | diabetes | 10 | 550 |
| 68 | [breast cancer](http://www.baidu.com/link?url=Q_xMJ6_7out6yd02aP6PpP_yAhkhgYpGu2d-uAX6_SAoMGk7jr_lfgn41AUl8AztkfPoNZSIMfd5N_nyAFUEmuKtzrgy-clVL67s386c5G4_WdX38n-g2rwP6B-K0qrQ) | 11 | 650 |
| 68 | [cervical disc](http://www.baidu.com/link?url=GGZ1jmIIessdVrP1FfMIiXVW9GRyazo5YMGq0NPqlty21IL5yCwdgc3cnIJYizEP8-b498yFg2BwFamTvOS7vuD6UryEUaP6NJDrWPKX4Zy6RlL_35UrLQY-BHxT4h8N) | 15 | 1069.79 |
| 69 | hypertension | 9 | 750 |
| 71 | [coronary heart disease](http://www.baidu.com/link?url=52nEjbM7Tuqz6LsuIIc0qJdmJV2LhCZWr2SXUPCNIVwu-BA-fEI4vNaxi1tzCVMPOwwfKRDANcKwvgq6v8G1FiTNLfWOO9r7AFBzjCNE65Ajzah_01FqPzavP55L9rTv)、[cataract](http://www.baidu.com/link?url=Vzprg_4TpcvSKFwCOn9FEeIN0nnQ_md5qHRcmY8Cp8dnApeZXaF1fZDIREm0q5LYdPjh14sIlovqum1kLDVz4Xg-jCK5nbF_CHcTz73jfeW)、 myeloneuritis | 14 | 912.5 |
| 68 | [hepatic cyst](http://www.baidu.com/link?url=LiHcvAhwbTFOZ6L3O_FH5a8StebLh_7laXiuVBG9W_sHZi2yj6th1_rSkrTX7EeQE5tBJXiusvxMjfLbsgzLUhgZ_CcuSfmXpKZFL53tL7hBzaUOvi8euepHXXx5GiOn) | 11 | 634 |
| 58 | hypertension | 8 | 912.5 |
| 72 | [bladder cancer](http://www.baidu.com/link?url=O95dCF2ckFQyo0BDPrYMz7J6UmngXcuZsJpk4AEScyKtw9133jRjHhPfpTQ7-EFxDSD_O45ONbx7k6zp0qL-nbhTO50kD4n1UrqIpzuwKt8TeSOmN1Hlif4C-nuJErBR) | 21 | 400 |
| 57 | hypertension | 4 | 600 |
| 55 | [coronary heart disease](http://www.baidu.com/link?url=52nEjbM7Tuqz6LsuIIc0qJdmJV2LhCZWr2SXUPCNIVwu-BA-fEI4vNaxi1tzCVMPOwwfKRDANcKwvgq6v8G1FiTNLfWOO9r7AFBzjCNE65Ajzah_01FqPzavP55L9rTv) | 11 | 825 |
| 64 | [Hepatitis B carriers](http://www.baidu.com/link?url=Vpa-jPVqNx3RbThPtA1u39A134Jgjh7a8WwBZp4JqngStL8bcjRK0U6W_-Yx6UKUu6DGFgYBBq6MbKWT980qIPohdu92kn_OqL2LMXmDKNlUDH-5tc3V70NYgBBiD0fm) | 7 | 625 |
| 44 | [gallbladder polyps](https://www.baidu.com/link?url=Cl6TCIigRr4mudPYsi8Ipe7N4i8g2pl1mxl_9BVsB_K_QuGDyolESaP2UzZEcOLgBhILSjvTBa4v-LFT6lgWeP6BLZppGgg4bo6KcdAn-ES_mp6PdogKk-VUjazJB3MS&wd=&eqid=c6ed3c8b001ad9d200000004602383a1) | 9 | 1050 |
| 68 | HBsAg, HBeAb and HBcAb positive、[encephalanalosis](https://www.baidu.com/link?url=Uh1g1aEdizfm64YfJpTv8q20iTjgSbQCwmovJ5eWz8iHN7vRIKBE8kg5kBTv2K6C-rtmpSfpqp-U8_nB6tAICNLmfjW7hPcelfL_ZNhwAKdmTBz3X5V9zvFfbjkZXdsh&wd=&eqid=d8554cba0000716c000000046023840f) | 8 | 750 |
| 68 | diabetes | 7 | 958.8 |
| 58 | lumbar and cervical spondylosis | 11 | 700 |

1. **Lobe and gyrus**

The 246 ROIs were grouped by 24 gyri that belongs to 7 lobes as follows:

**Table 4.** The relationship between gyrus and lobe in this revision

| Lobe |  | Gyrus | ID start-end |
| --- | --- | --- | --- |
| Frontal Lobe | 1 | SFG, Superior Frontal Gyrus | 1-14 |
|  | 2 | MFG, Middle Frontal Gyrus | 15-28 |
|  | 3 | IFG, Inferior Frontal Gyrus | 29-40 |
|  | 4 | OrG, Orbital Gyrus | 41-52 |
|  | 5 | PrG, Precentral Gyrus | 53-64 |
|  | 6 | PCL, Paracentral Lobule | 65-68 |
| Temporal Lobe | 7 | STG, Superior Temporal Gyrus | 69-80 |
|  | 8 | MTG, Middle Temporal Gyrus | 81-88 |
|  | 9 | ITG, Inferior Temporal Gyrus | 89-102 |
|  | 10 | FuG, Fusiform Gyrus | 103-108 |
|  | 11 | PhG, Parahippocampal Gyrus | 109-120 |
|  | 12 | pSTS, posterior Superior Temporal Sulcus | 121-124 |
| Parietal Lobe | 13 | SPL, Superior Parietal Lobule | 125-134 |
|  | 14 | IPL, Inferior Parietal Lobule | 135-146 |
|  | 15 | Pcun, Precuneus | 147-154 |
|  | 16 | PoG, Postcentral Gyrus | 155-162 |
| Insular Lobe | 17 | INS, Insular Gyrus | 163-174 |
| Limbic Lobe | 18 | CG, Cingulate Gyrus | 175-188 |
| Occipital Lobe | 19 | MVOcC, MedioVentral Occipital Cortex | 189-198 |
|  | 20 | LOcC, lateral Occipital Cortex | 199-210 |
| Subcortical Nuclei | 21 | Amyg, Amygdala | 211-214 |
|  | 22 | Hipp, Hippocampus | 215-218 |
|  | 23 | BG, Basal Ganglia | 219-230 |
|  | 24 | Tha, Thalamus | 231-246 |

1. **Prediction in different motor symptoms**

To study if the results would be different across different symptoms, we divided the overall UPDRS-III score according to different symptoms. The prediction results of the four networks in the improvement rate of gait and hand movement symptoms compared with overall symptoms were shown in Table 5. These results showed that RH network is most predictive on all of these symptoms.

**Table 5.** The prediction results of the four networks in the improvement rate of gait and hand movement symptoms compared with overall symptoms

| LH | RH | Ho | He |
| --- | --- | --- | --- |
| (r=0.15, p=0.29) | (r=0.26, p=0.05) | (r=0.21, p=0.13) | (r=0.12, p=0.39) |

1. **Gait**

| LH | RH | Ho | He |
| --- | --- | --- | --- |
| (r=0.17, p=0.24) | (r=0.30, p=0.03) | (r=0.17, p=0.21) | (r=0.06, p=0.67) |

**b) Hand_movement**

| LH | RH | Ho | He |
| --- | --- | --- | --- |
| (r=0.15, p=0.29) | (r=0.37, p=5.68E-03) | (r=0.29, p=3.72E-02) | (r=0.08, p=0.57) |

1. **Overall**
